# Prolonged treatment with Y-27632 promotes the senescence of primary human dermal fibroblasts by increasing the expression of IGFBP-5 and transforming them into a CAF-like phenotype

**DOI:** 10.18632/aging.103910

**Published:** 2020-08-25

**Authors:** Xiangyong Li, Qian Zhou, Shuangshuang Wang, Ping Wang, Juan Li, Zhiwei Xie, Chang Liu, Jie Wen, Xunwei Wu

**Affiliations:** 1 Department of Tissue Engineering and Regeneration, School and Hospital of Stomatology, Cheeloo College of Medicine, Shandong University and Shandong Key Laboratory of Oral Tissue Regeneration and Shandong Engineering Laboratory for Dental Materials and Oral Tissue Regeneration, Jinan, China; 2 Key Laboratory of Biotechnology and Biological Resource Utilization in Universities of Shandong and College of Life Science, Dezhou University, Dezhou, China; 3 Key Laboratory of Chemical Biology (Ministry of Education), School of Pharmaceutical Sciences, Shandong University, Jinan, China; 4 Department of Outpatient Surgery, Qilu Hospital, Shandong University, Jinan, Shandong, China; 5 Department of Stomatology, Shengli Oilfield Central Hospital, Dongying, Shandong, China

**Keywords:** Rho kinase inhibitor, Y-27632, senescence, IGFBP-5, dermal fibroblast

## Abstract

The Rho-kinases (ROCK) inhibitor Y-27632 has been shown to promote the growth of epidermal cells, however, its potential effects on human dermal fibroblasts (HDFs) need to be clarified. Here we show that prolonged treatment of HDFs with Y-27632 decreased their growth by inducing senescence, which was associated with induction of the senescence markers p16 and p21, and downmodulation of the ERK pathways. The senescent HDFs induced by Y-27632 acquired a cancer-associated-fibroblast (CAF)-like phenotype to promote squamous cell carcinoma (SCC) cell growth *in vitro*. Expression of a newly identified target of Y-27632 by RNA-seq, insulin growth factor binding protein 5 (IGFBP-5), was dramatically increased after 24 h of treatment with Y-27632. Adding recombinant IGFBP-5 protein to the culture medium produced similar phenotypes of HDFs as did treatment with Y-27632, and knockdown of IGFBP-5 blocked the Y-27632-induced senescence. Furthermore, Y-27632 induced the expression of an IGFBP-5 upstream gene, GATA4, and knockdown of GATA4 also reduced the Y-27632-induced senescence. In summary, these results demonstrate for the first time that Y-27632 promotes cellular senescence in primary HDFs by inducing the expression of IGFBP-5 and that prolonged treatment with Y-27632 potentially transforms primary HDFs into CAF-like cells.

## INTRODUCTION

Rho-kinases (ROCK), including ROCK1 and ROCK2, are signal transducers that connect cell membrane receptors to cytoskeletons [1]. ROCKs impact numerous cellular functions and are involved in multiple biological processes [2–4]. ROCK inhibitors have been proposed for a number of therapeutic applications such as hypertension [5–8], ischemic stroke [9], cancer [10–12], erectile dysfunction [13], glaucoma [14], multiple sclerosis [15] and spinal cord injury [16, 17]. Notably, a ROCK inhibitor, Fasudil, has been approved to use clinically for the treatment of pulmonary hypertension in Japan and China. The short-term efficacy and safety of ROCK inhibitors has been evaluated to be effective without apparent side effects, however the long-term efficacy and safety of ROCK inhibitors has not been addressed [18]. Therefore, it will also be important to explore the effects of prolonged treatment with ROCK inhibitors on cells.

Y-27632, a small molecule compound, originally was identified as a relaxant of vascular and bronchial smooth muscles [19], and can inhibit both ROCK1 and ROCK2 [20]. Y-27632 has been most widely used to study ROCK functions *in vitro* and *in vivo* [11, 21–27]. The biological functions of Y-27632 have already been demonstrated to be cell or cell content specific [28]. For example, Y-27632 was reported to inhibit mouse B16 melanoma cell growth [29], but we recently reported that Y-27632 actually enhances the growth of BRAF-mutant human melanoma cells [30]. Y-27632 has been reported to promote the proliferation and migration of keratinocytes [23, 31]. Interestingly, when we cultured a mixture of epidermal cells and dermal cells from the skin in the presence of Y-27632, we found that the growth of epidermal cells was significantly increased but the growth of dermal cells was clearly inhibited [32], which was inconsistent with a report by Piltti et al. showing that Y-27632 increased the proliferation of dermal fibroblasts [32, 33]. Therefore, the detailed role of Y-27632 on the growth of skin dermal fibroblasts needs to be further clarified. Moreover, it will also be important to understand its underlying molecular mechanism of action of Y-27632 on fibroblasts that will be pivotal to any clinical application.

Changes of stroma cells, such as fibroblasts, have been shown to play critical roles in tumor initiation and development [34, 35]. Activated fibroblasts, which are called cancer-associated fibroblasts (CAFs), are the main component of the tumor microenvironment [36]. A large number of studies have demonstrated that CAFs promote tumor growth and metastasis [37–40]. It has been demonstrated that senescent fibroblasts are capable of releasing cytokines and growth factors into their microenvironment, termed the “senescence-associated secretory phenotype (SASP)”, which turns senescent fibroblasts into CAF cells [41]. Therefore, it will be interesting to investigate whether ROCK inhibitors potentially promote the senescence of dermal fibroblasts to gain a CAF phenotype.

The goal of the present study was to investigate the detailed effects of Y-27632 on the growth of primary human dermal fibroblasts (HDFs) with prolonged treatment to determine whether it promotes or inhibits growth through the regulation of cellular senescence and to explore the molecular mechanisms underlying that process.

## RESULTS

### Prolonged treatment with Y-27632 inhibits the growth of HDFs

**Figure 1:**
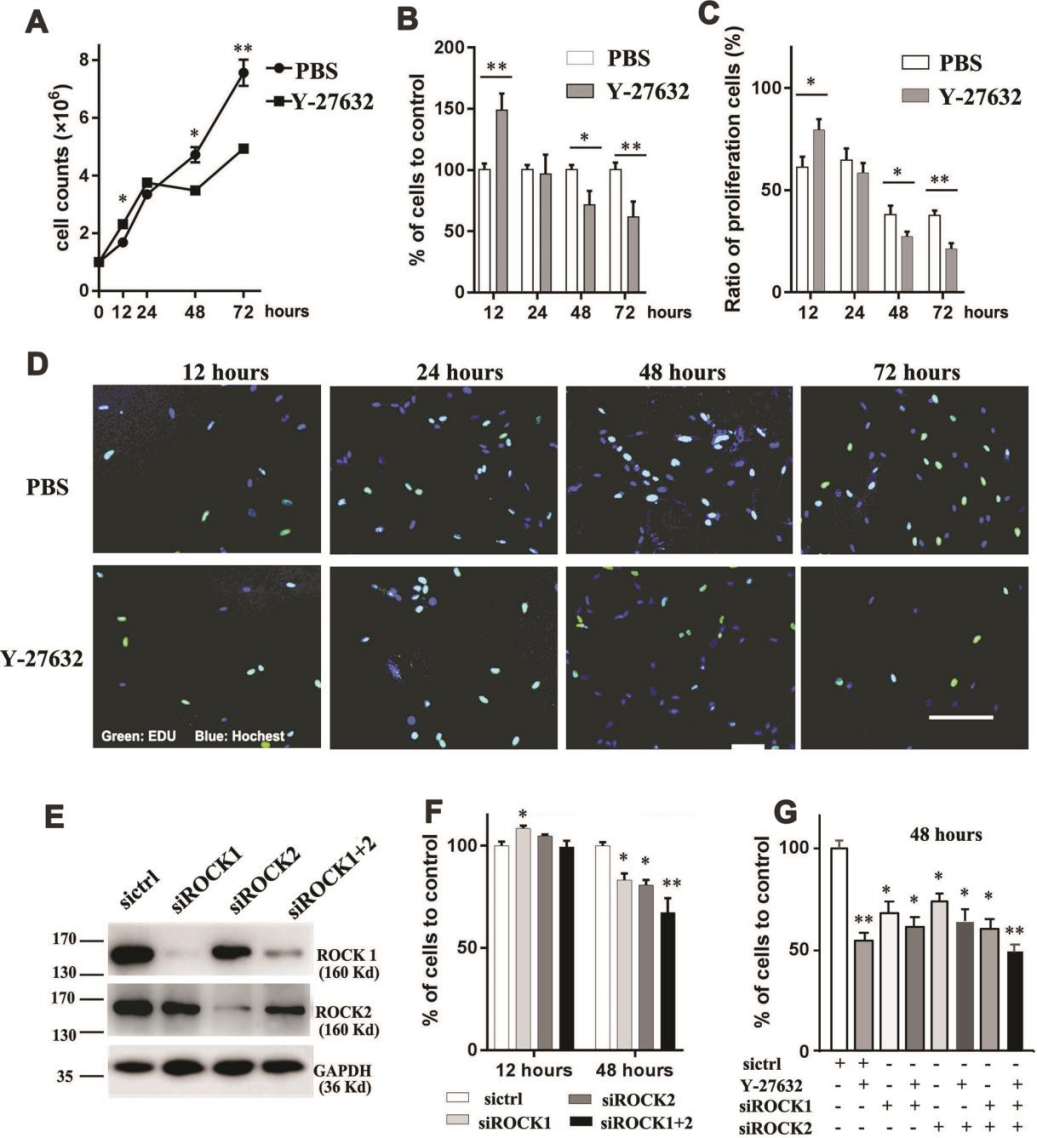
**Inhibition of ROCK signaling decreases the growth of HDFs.** (**A**) One x 10^6^ HDFs were seeded in growth medium in the presence of 10 μM Y-27632 or PBS (as control); cells were then collected at the indicated times to analyze cell numbers using an automatic cell-counter; (**B**) HDFs were cultured under the same conditions as in (**A**); cells were collected at the indicated times to analyze cell proliferation using a CCK8 kit, and the percentage of cell growth was calculated relative to the corresponding control (PBS) as 100. (**C**) and (**D**): HDFs were cultured with 10 μM Y-27632 or control PBS, then fixed at the indicated times and stained with the proliferation marker EdU (green) and DAPI to stain nuclei (blue). The quantification of EdU-positive cells is shown in (**C**), which was calculated as the percentage of green cells (total count of 200 cells) of DAPI positive cells (blue); representative images of EdU staining are shown in (**D**). Scale bars = 100 μm. (**E**) HDFs were transfected with siRNAs specific for ROCK1 (siROCK1), ROCK2 (siROCK2) or both ROCK1 and ROCK2 (siROCK1+2); control HDFs were transfected with a scrambled siRNA (sictrl), and cells were collected at 48 h after transfection to detect the expression of ROCK1 and ROCK2 by western-blot (**E**). (**F**) One x 10^6^ HDFs were transfected with siRNAs specific for ROCK1 and/or ROCK2 and a scrambled siRNA (sictrl), at 12 and 48 h after transfection cells were collected to analyze cell proliferation using a CCK-8 kit. The percentage of cells was calculated relative to cells transfected with the negative control siRNA as 100. (**G**) One x 10^6^ HDFs were transfected with siRNAs specific for ROCK1 and/or ROCK2 and a scrambled siRNA (sictrl) plus/minus Y-27632 and at 48 h after transfection, cells were collected to analyze cell proliferation using a CCK-8 kit. The percentage of cells was calculated relative to cells transfected with the negative control siRNA without treatment of Y-27632 as 100. All experiments were repeated at least 4 times. *P<0.05, **P<0.01, when two groups were compared as indicated, or were compared to the corresponding controls.

In order to elucidate the effects of Y-27632 on dermal cell growth, we cultured HDFs in the absence or presence of 10 μM Y-27632 for 12 to 72 h. As shown in Figure 1A, during the first 24 h of culture, Y-27632 treated HDFs grew faster than the PBS treated controls, however after that time, their growth was significantly inhibited compared to the PBS controls (Figure 1A). To further confirm that prolonged treatment with Y-27632 inhibits the growth of HDFs, a CCK8 cell counting assay was carried out with the same results (Figure 1B). The effect of Y-27632 on HDFs was quantified using EdU to stain proliferating cells. Figure 1D shows EdU-positive cells (green) at different time points with quantification of those results shown in Figure 1C. Again, the results confirmed that there are more proliferating HDFs at 12 h of Y-27632 treatment compared to PBS treated controls, and significantly fewer HDFs at 48 and 72 h (Figure 1C, 1D). In order to validate the effects of Y-27632 on HDFs derived in our laboratory, we treated commercial HDFs (cHDFs) in the same manner and obtained similar results (Supplementary Figure 1).

Next we determined whether the growth inhibition of HDFs after prolonged treatment with Y-27632 occurred through the ROCK pathway. To test that, we knocked down the expression of genes encoding ROCK proteins by transfecting HDFs either with ROCK1 siRNA (siROCK1) or ROCK2 siRNA(siROCK2) or with both ROCK1 and ROCk2 siRNAs (siROCK1+2) or a scramble siRNA (sictrl). RT-qPCR and western-blot analyses showed that the expression levels of ROCK1 and ROCK2 decreased significantly after RNA interference (Supplementary Figure 2 and Figure 1E). The growth of HDFs after 48 h, as determined by CCK8 staining, was significantly decreased in the ROCK knockdown groups compared to the control group (sictrl) (Figure 1F). To investigate potential off-targets of Y-27632 that might affect the growth of HDFs, we combined Y-27632 treatment with knockdown of ROCK1/2 in HDFs. We found that treatment with Y-27632 didn’t significantly further enhance the inhibition of HDF growth compared with the knockdown of ROCK1/2 (Figure 1G). Taken together, these data suggest that the prolonged treatment of Y-27632 blocks the growth of HDFs mainly through the inhibition of ROCK function *in vitro*.

### Prolonged inhibition of ROCK activity promotes the senescence of HDFs

**Figure 2:**
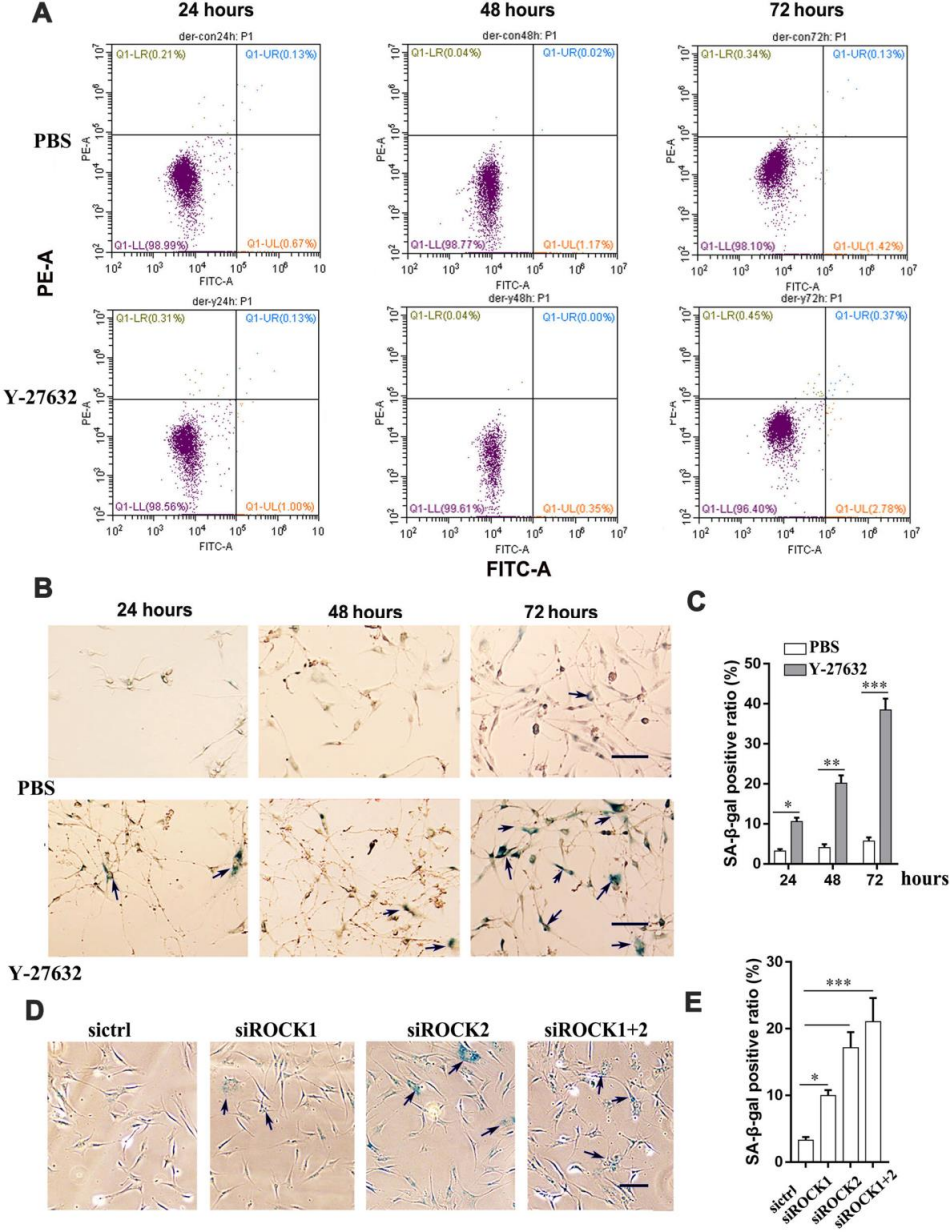
**The inhibition of ROCK signaling promotes the cellular senescence of HDFs.** (**A**) HDFs were treated with PBS or 10 μM Y-27632, then were collected at the indicated times, stained with annexin V-FITC combined with PI, then analyzed by FACS for apoptotic cells. (**B**) HDFs were treated with PBS (upper) or 10 μM Y-27632 (lower), then fixed at the indicated times and analyzed using a SA-β-gal staining kit for senescent cells. scale bar = 100 μm. (**C**) Quantification of (**B**), the percentage of SA-β-gal positive cells (blue, arrows) was calculated based on counting a total of 500 cells. (**D**) HDFs were transfected with siRNAs for ROCK1 and/or ROCK2, or with a scrambled siRNA as a control (sictrl), and at 48 h after transfection, cells were fixed and stained with SA-β-gal for senescent cells (blue, arrows), scale bar = 100 μm. (**E**) Quantification of SA-β-gal-positive cells in (**D**), the percentage of SA-β-gal-positive cells was calculated based on counting a total of 500 cells. All experiments were performed at least 3 times,*P<0.05, **P<0.01, ***P<0.005 when two groups were compared as indicated or were compared to the corresponding controls.

Next, we determined whether the inhibited growth of HDFs elicited by Y-27632 is due to an increase of cell death. First, we checked whether Y-27632 induces apoptosis in HDFs. The percentage of apoptotic cells (i.e. FITC- and PI-positive cells) did not change significantly between the control (PBS) and the Y-27632 treated groups at 24, 48 and 72 h after seeding (Figure 2A). Next, we tested whether Y-27632 promoted cellular senescence in HDFs. Using SA-β-gal staining to detect cellular senescence, we found that Y-27632 treatment did indeed increase the number of SA-β-gal-positive cells (arrows), i.e. senescent cells, at 24, 48 and 72 h after seeding (Figure 2B, 2C). Increased positive staining for SA-β-gal activity (arrows, Figure 2D) was also found in HDFs after knockdown of ROCK1/ROCK2 genes by siRNAs (Figure 2D, 2E). A similar result on cellular senescence elicited in commercial HDFs by Y-27632 was also confirmed (Supplementary Figure 3A, 3B). We further determined whether Y-27632 enhances cellular senescence by analyzing expression of the senescence marker Lamin B1 [42] and found that treatment with Y-27632 decreased Lamin B1 expression both in HDFs and in cHDFs (Supplementary Figure 3C). Taken together, these data suggest that Y-27632 enhances the senescence of HDFs, which results in the inhibition of their growth.

### Prolonged treatment with Y-27632 promotes the transformation of HDFs to a CAF phenotype

**Figure 3:**
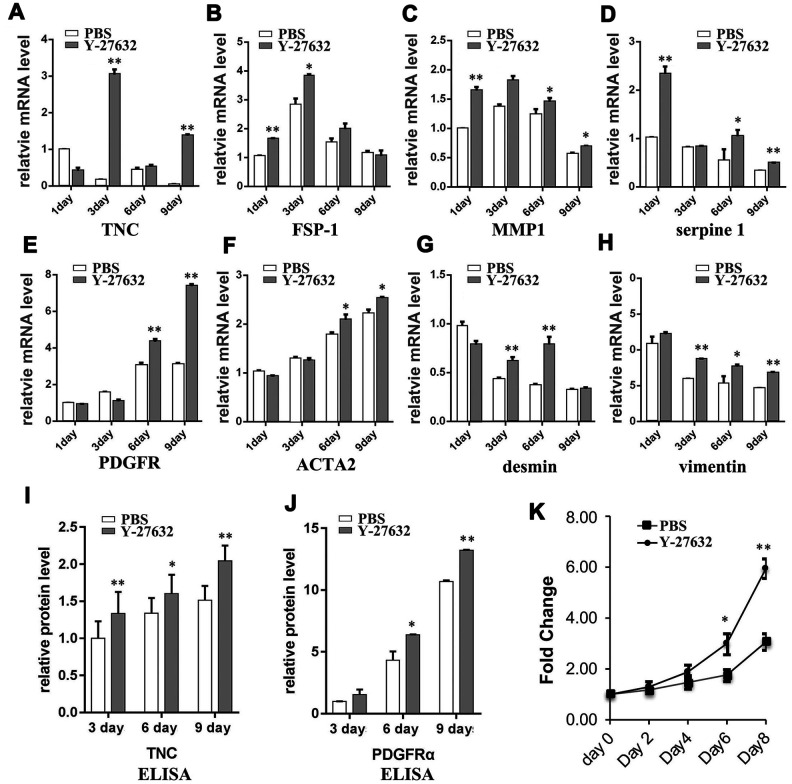
**Prolonged treatment of HDFs with Y-27632 causes a CAF-like phenotype.** (**A**–**H**) HDFs were treated with PBS or 10 μM Y-27632 for 1, 3, 6 and 9 days. HDFs were collected at the indicated times, and mRNA expression levels of several cancer associated fibroblast phenotype related genes as indicated were analyzed using RT-qPCR. The fold changes of these genes in Y-27632 treated HDFs relative to the control (expression level as 1) are shown. (**I**) and (**J**) HDFs were treated as described for (**A**–**H**), and cell culture media were collected at the indicated times for ELISA assays of protein levels of TNC (**i**) and PDFGRα (**J**). The fold changes of TNC and PDFGRα in Y-27632 treated HDFs relative to the control at 3 days (expression level as 1) are shown. (**K**) HDFs were cultured in epidermal medium with PBS or 10 μM Y-27632 for 3 days, then the medium was replaced without adding Y-27632 for another 3 days, after which the conditioned media were collected and used to culture SCC13 cells. The SCC cells were cultured with the conditioned media for 8 days, and then were assessed every two days for the analysis of cell growth using a CCK8 kit. All experiments were repeated at least 4 times, *P<0.05, **P<0.01 when compared to the corresponding control.

The senescence of stromal cells has been shown to be associated with the induction of CAF effector genes. In order to further investigate the behavior of senescent HDFs induced by ROCK inhibitors, we extended the treatment time of HDFs with Y-27632 to a much longer time (9 days), then collected these cells at different time points to analyze the expression of known CAF effector genes. RT-qPCR analysis showed that treatment with Y-27632 enhanced the mRNA expression levels of TNC, FSP-1, MMP1, serpine1, PDGFR, ACTA2, desmin and vimentin, a typical CAF expression pattern (Figure 3A–3H).

To further test whether the senescent HDFs induced by Y-27632 could secrete these CAF proteins, we collected conditioned media at different time points from HDFs treated with Y-27632 and measured the media for levels of TNC and PDGFRα proteins (Figure 3I, 3J). To verify the hypothesis that Y-27632-treated HDFs have the potential to transform into a CAF-like phenotype, HDFs were cultured in the presence of Y-27632 for 3 days to induce senescence (cells cultured without Y-27632 were used as a control). They were then placed in medium without Y-27632, and after another 3 days of culture, the conditioned media were collected and used to treat cultures of SCC13 human skin squamous cell carcinoma cells. The results showed that the conditioned media derived from HDFs treated with Y-27632 significantly promoted the growth of SCC cells when compared to the control medium derived from cells without treatment by Y-27632 (Figure 3K). Taken together, these data suggest that senescent HDFs induced by prolonged treatment with Y-27632 acquire a senescence-associated secretory phenotype (SASP).

### Inhibition of ROCK signaling in HDFs at early time points increases the expression of the proliferation marker cyclinD1 and the senescence markers p16 and p21

**Figure 4:**
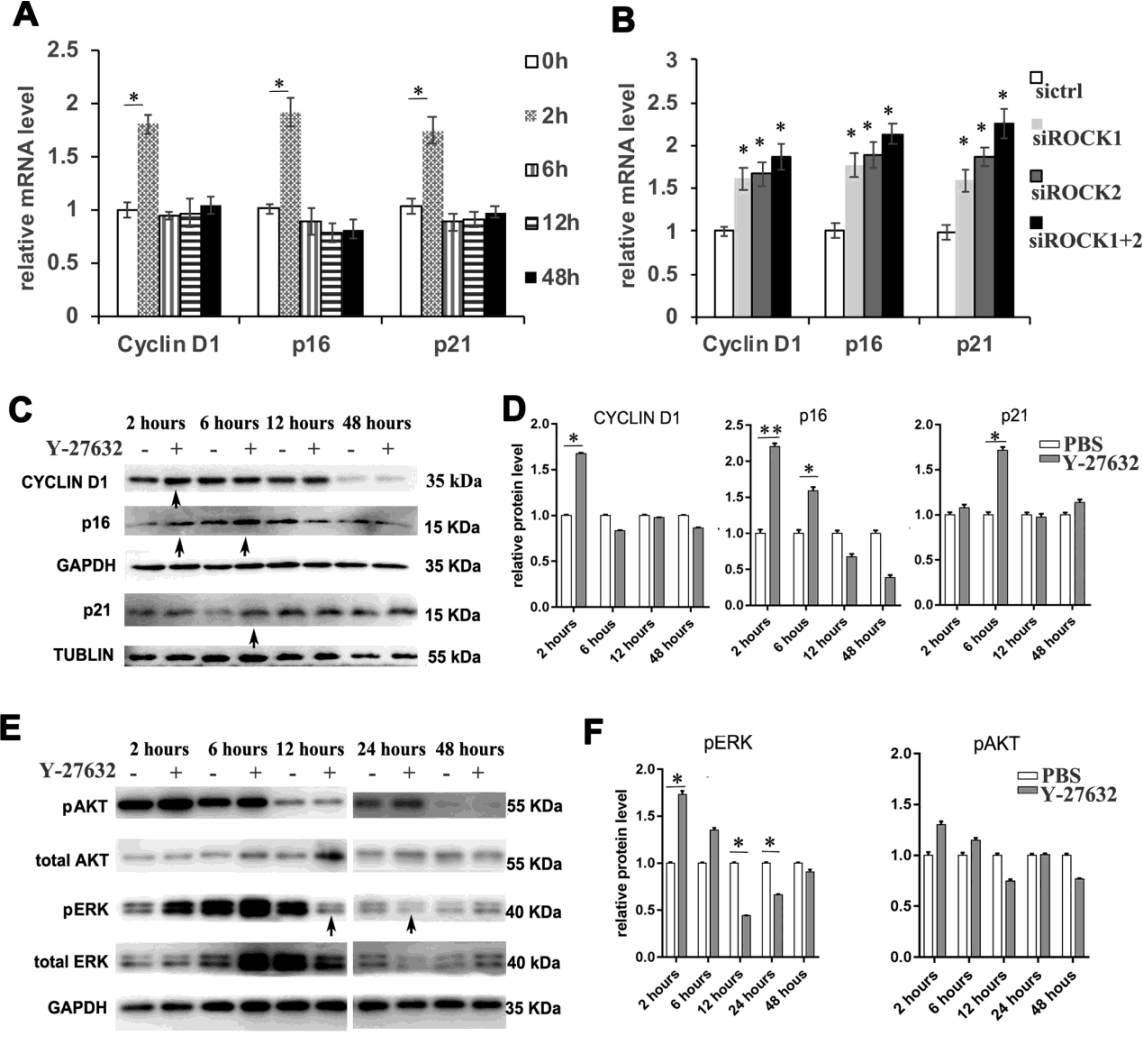
**Inhibition of ROCK signaling increases the expression of proliferation and senescence markers.** (**A**) HDFs were cultured in growth medium in the presence of PBS or 10 μM Y-27632, then were collected at the indicated times for RT-qPCR analysis of cyclin D1, p16 and p21. The expression level of those 3 genes was adjusted with the house-keeping gene 36B4, the mRNA level in the Y-27632 treated HDFs relative to the control HDFs (expression level as 1) is shown. (**B**) HDFs were transfected with siRNAs targeting ROCK1 or and ROCK2, the control HDFs were transfected with a scrambled siRNA (sictrl). HDFs were collected at 48 h after the transfection and subjected to RT-qPCR analysis to detect mRNA expression levels of cyclin D1, p16 and p21. The relative mRNA level of 3 genes in ROCK knockdown HDFs is shown. (**C**, **D**) HDFs were treated with PBS (-) or 10 μM Y-27632 (+), then were collected at the indicated times and cellular lysates were analyzed for protein expression levels of cyclin D1, p16 and p21 by western blot. GAPDH or β-tubulin protein levels were used as loading controls. Arrows inidcated increased expression of genes in the Y-27632 treated cells (**C**). The fold change of those proteins in the Y-27632 treated HDFs relative to the control HDFs (expression level as 1) is shown on the right (**D**). (**E**, **F**) Western blot analysis of pERK and pAKT in HDFs at the indicated times; GAPDH protein levels were detected as a loading control. The fold change of these 3 proteins in the Y-27632 treated HDFs relative to the control HDFs (expression level as 1) is shown on the right (**F**). Densitometry measurements for pERK and pAKT were normalized to the amounts of total ERK and AKT, respectively. Arrows inidcated decreased expression of phorylation form of ERK in the Y-27632 treated cells (**E**). All experiments were repeated at least 3 times, *P<0.05, **P<0.01 when two groups were compared as indicated, or were compared to the corresponding controls.

To further understand the effect of ROCK inhibition on the growth of HDFs, we studied the expression of cyclinD1, p16 and p21. RT-qPCR revealed that Y-27632 induces the expression of both the p16 and p21 genes, classic markers of cellular senescence, at early time points of treatment (Figure 4A). Those expression patterns of p16 and p21 were also found in Y-27632 treated cHDFs (Supplementary Figure 4A, 4B). Interestingly, Y-27632 also increased the expression of the proliferation marker cyclin D1 (Figure 4A). Consistent results were found with the siRNA mediated knockdown of ROCK gene expression (Figure 4B). These results were further confirmed by western-blot analysis (arrows, Figure 4C) and its corresponding quantification data (Figure 4D). Since HDFs treated with Y-27632 showed increased expression of cyclin D1, we tested if the classic pathways regulating cell proliferation and survival, i.e. the ERK and AKT pathways, are also affected. As shown in Figure 4E, 4F, the levels of phosphorylated ERK1/2 (pERK) were increased at earlier time points of treatment with Y-27632, but then appeared to significantly decrease (12h, 24h, arrows, Figure 4E, 4F). For the AKT activity, phosphorylated AKT also slightly increased at early time points of Y-27632 treatment, and decreased after 12 h, but these changes were not statistically significant (Figure 4E, 4F). Taken together, these data suggest that Y-27632 activates both the proliferation and the senescence pathways in HDFs at early time points of treatment, and corresponding changes of the ERK pathway were involved.

### RNA-seq analysis identifies IGFBP-5 as a potential factor in the action of Y-27632

The above data suggested that Y-27632 plays a role in regulating cell proliferation and senescence. To further understand its molecular mechanisms, we performed RNA-seq analysis of HDFs at 12 h and 48 h with or without Y-27632 treatment. The RNA-seq analysis revealed that 69 and 239 DEGs were identified between the untreated control (PBS) and the Y-27632 treated group at 12 h and 48 h, respectively, and among all those DEGs, 36 genes appeared to be differently expressed at both the 12 h and 48 h time points (Supplementary Figure 5A). The expression profiles of all 272 DEGs were visualized with a heatmap (Supplementary Figure 5B), which showed that there were four mainly separated sample subclusters in the hierarchy structure of clusters (Supplementary Figure 5B). Specifically, considering the regulation direction of the DEGs, genes in cluster 1 were downregulated after 12 h of Y-27632 treatment, genes in cluster 2 were upregulated after 12 h or 48 h of treatment, genes in cluster 3 were up-regulated after treatment for 48 h, while genes in cluster 4 were downregulated after 48 h of treatment. GO analysis revealed that genes in clusters 2 and 4 were highly enriched in the regulation of system process, negative regulation of growth. The distribution of all 4 groups in the 3-dimensional space projected by principal component analysis (PCA) of the expression profiles of the 272 DEGs was analyzed. The control and Y-27632 treated groups appeared separately along the second axis and were separated from one another, which verified this observation with a view toward group separation (Supplementary Figure 5C).

**Figure 5:**
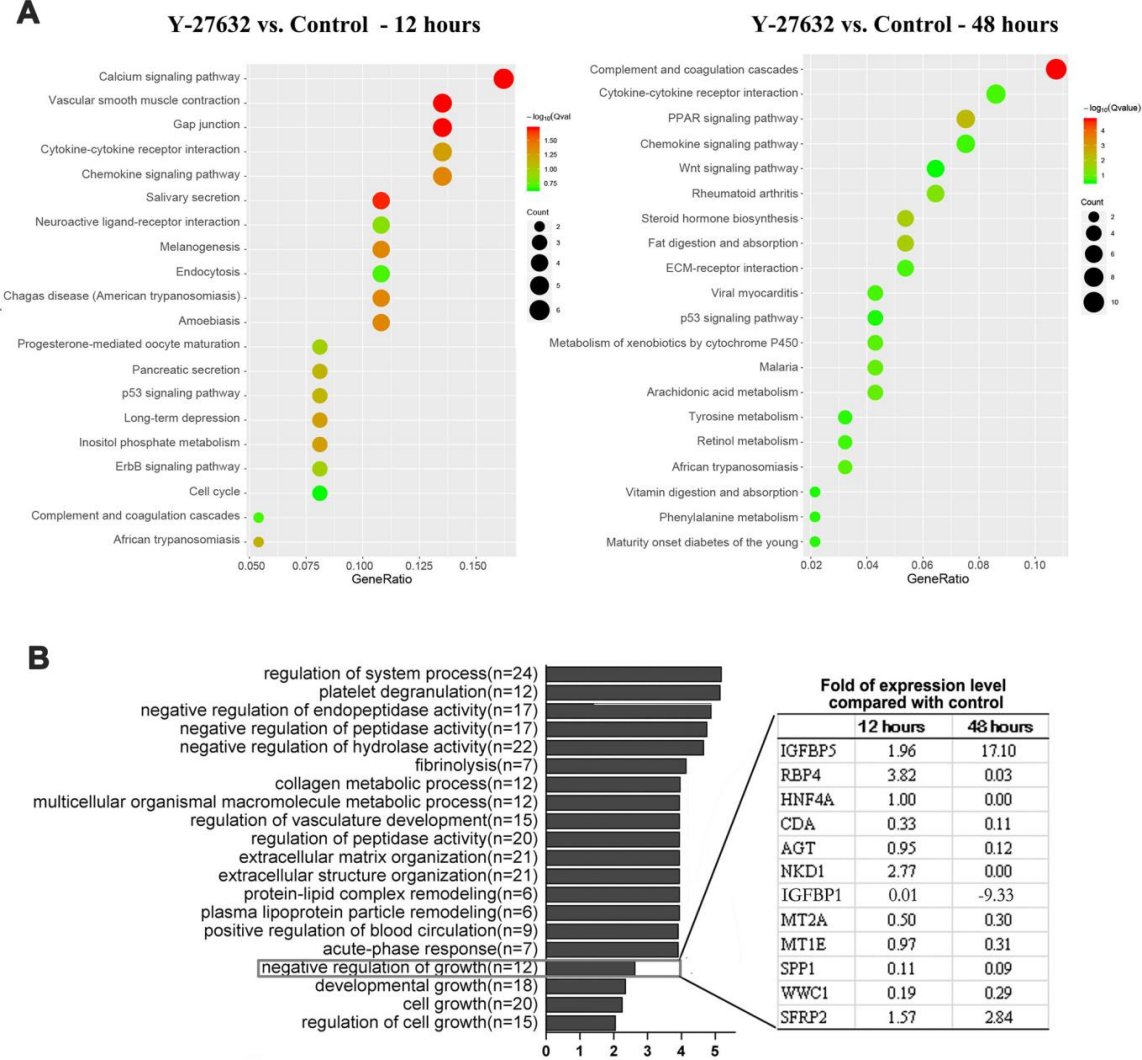
**Identification of IGFBP-5 as a potential factor in the decreased growth of HDFs treated with Y-27632.** (**A**, **B**) HDFs were cultured in growth medium in the presence of 10 μM Y-27632 or PBS (as a control) for 12 and 48 h, then were collected for the isolation of total RNAs for RNA-seq. KEGG pathway enrichment analysis of DEGs, and the top 20 pathways are shown for each time point (left panel for 12 h, right panel for 48 h) in (**A**). The size of each dot represents the degree of enrichment and the color indicates the degree of statistical significance. A Biological Process map of GO enrichment analysis is shown in (**B**). Genes related with ‘negative regulation growth’ from Gene Ontology (GO) enrichment analysis are listed in the table expanded on the right.

**Figure 6:**
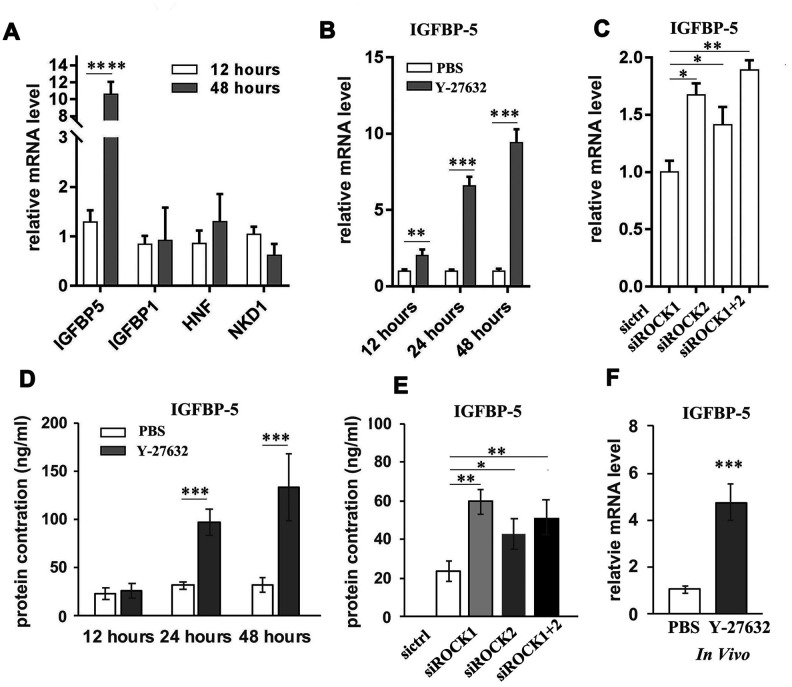
**Y-27632 induces expression of IGFBP-5 in HDFs *in vitro* and *in vivo*.** (**A**) Genes whose expression levels changed differently at 12 h and 48 h in (Figure 4) were detected by RT-qPCR analysis. Fold mRNA expression levels were normalized by the control at the indicated times. (**B**) HDFs were treated with PBS and 10 μM (y+) Y-27632 at the indicated times and total RNAs were extracted for RT-qPCR analysis of IGFBP-5. The fold change of IGFBP-5 relative to the control (expression level as 1) is shown. (**C**) HDFs were transfected with siRNAs specific for ROCK1 and/or ROCK2, with a scrambled siRNA as a control, and at 48 h after transfection, the mRNA expression level of IGFBP-5 was determined by RT-qPCR analysis. The fold change of IGFBP-5 in ROCK knockdown cells relative to the control (expression level as 1) is shown. (**D**) HDFs were treated with PBS or 10 μM (y+) Y-27632 and the conditioned medium was collected at the indicated times for ELISA analysis of IGFBP-5 protein level. (**E**) HDFs were transfected with siRNAs specific for ROCK1 and/or ROCK2, with a scrambled siRNA as a control, and at 48 h after transfection, the cells were replaced with fresh medium and cultured for another 48 hours to collect the conditioned medium for ELISA analysis of IGFBP-5 protein level. (**F**) The reconstituted skin formed from grafting HDFs together with human epidermal cells were collected after treatment with 10 μM Y-27632 or PBS for 2 weeks and total RNAs were extracted from the dissected dermis of the skin for RT-qPCR analysis of human IGFBP-5. All experiments were repeated at least 3 times. * P<0.05,**P<0.01, *** P<0.005 when compared to the control.

KEGG pathway analysis was performed to identify changed pathways for DEGs from 12 h to 48 h treatment of Y-27632, and the results showed that several signaling pathways related to cell growth and cellular senescence, such as cytokine, chemokine and p53 signaling pathways, were upregulated in the Y-27632 treated group at both time points (Figure 5A).We further sequenced 12 of the 239 DEGs at 48 h after Y-27632 treatment and identified genes that by GO analysis are negative regulators of cell growth (Figure 5B). Of those 12 genes in these two processes, the expression levels of 4 genes (IGFBP1, IGFBP-5, HNF4A and NKD1) changed significantly at both 12 h and 48 h of Y-27632 treatment. These results were validated by RT-qPCR analysis (Figure 6A), which showed that the expression of IGFBP-5 increased by 1.5-fold after 12 h of treatment with Y-27632, and increased more than 10-fold after 48 h of treatment. However, expression of the other 3 genes didn’t change significantly after Y-27632 treatment (Figure 6A). This effect of increased expression of IGFBP-5 elicited by Y-27632 was replicated using different HDFs derived from different donors, and was confirmed using cHDFs (Supplementary Figure 6A).

Again, we found that Y-27632 increased mRNA levels of IGFBP-5 in HDFs as early as at 12 h after treatment, with enhanced expression after 24 h (Figure 6B). The inhibition of ROCK by siRNA mediated knockdown also increased IGFBP-5 expression (Figure 6C). Since IGFBP-5 is a secreted protein that binds to insulin growth factors (IGFs), we next checked the protein level of IGFBP-5 in conditioned media collected from cultures of HDFs at different time points. We found that treatment with Y-27632 increased the protein level of IGFBP-5 in the culture medium (Figure 6D) and further, found that the culture media of ROCK knockdown HDFs also showed increased levels of IGFBP-5 (Figure 6E). To validate that Y-27632 enhances the expression of IGFBP-5 in HDFs *in vivo*, human skin reconstituted from grafting cultured HDFs together with human epidermal cells, was subcutaneously injected with 10 μM Y-27632 for 2 weeks. The reconstituted skin was then collected and dissected under a microscope to carefully separate the dermis from the epidermis, after which the dermis was processed to extract RNA for RT-PCR analysis of IGFBP-5 expression. We found that treatment with Y-27632 significantly induced IGFBP-5 expression dermal cells *in vivo* (Figure 6F). In summary, blocking ROCK function in HDFs significantly induces the expression, production and secretion of IGFBP-5 *in vitro* and likely also enhances its expression *in vivo*.

### Y-27632 promotes cell senescence in HDFs by regulating the expression of IGFBP-5

**Figure 7:**
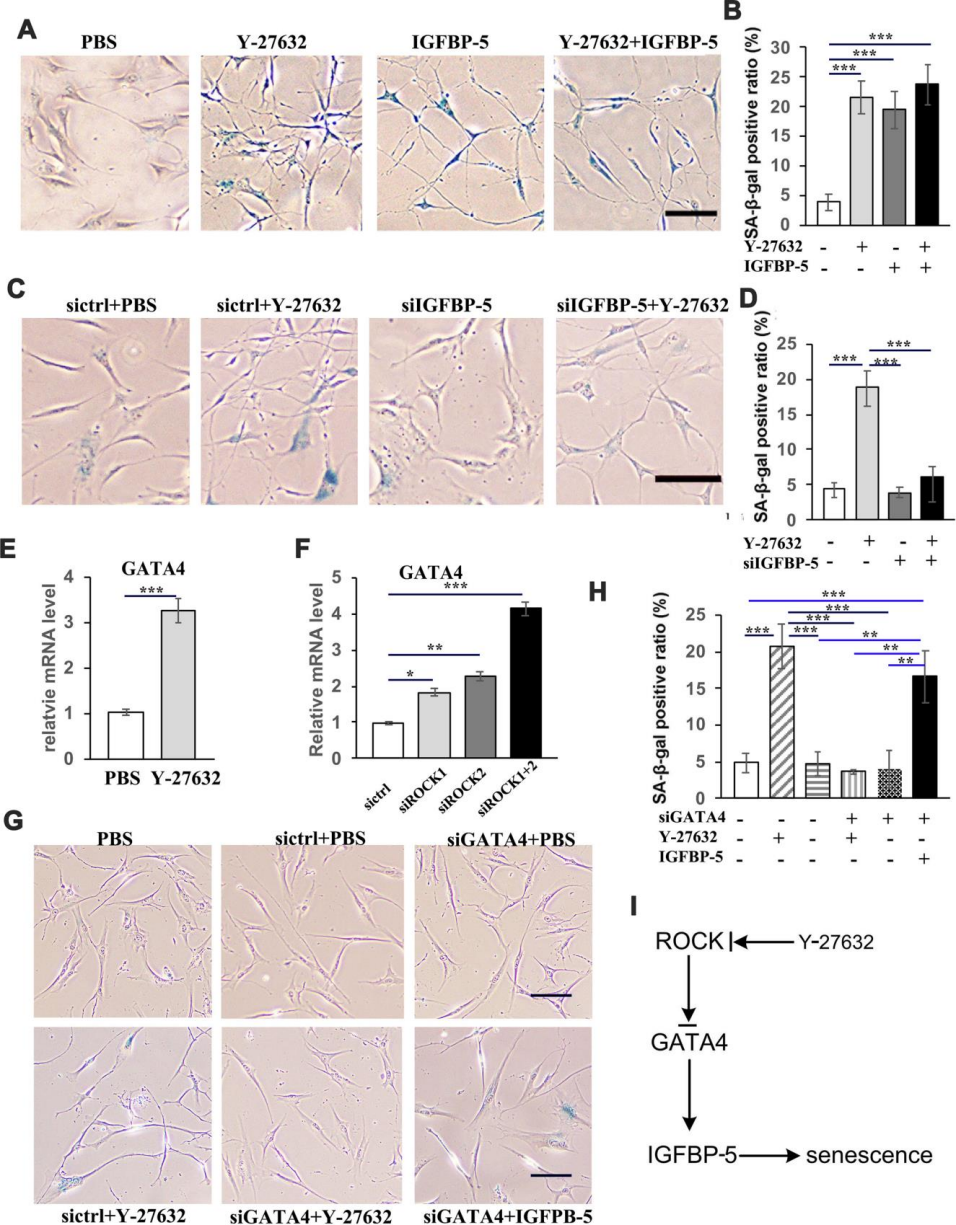
**Y-27632 promotes the senescence of HDFs through regulation of GATA4/IGFBP-5.** (**A**) HDFs were treated with or without 10 μM Y-27632 or with or without 100 ng/ml IGFBP-5 for 48 h, then were fixed and analyzed using a SA-β-gal staining kit to detect senescent cells. scale bar = 100 μm. (**B**) Quantification of SA-β-gal-positive cells in (d), the percentage of SA-β-gal-positive cells was calculated based on counting a total of 500 cells. (**C**) HDFs were transfected with a siRNA targeting IGFBP-5 (siIGFBP-5) or a scrambled siRNA (sictrl). After transfection for 24 h, HDFs were treated with PBS or 10 μM Y-27632 for 48 h and were then fixed and analyzed using a SA-β-gal staining kit to detect senescent cells. scale bar = 100 μm. (**D**) Quantification of SA-β-gal-positive cells in (**C**), the percentage of SA-β-gal-positive cells was calculated based on counting a total of 500 cells. (**E**) HDFs were cultured in growth medium in the presence of PBS or 10 μM Y-27632, then collected at 48 h after treatment for RT-qPCR analysis of GATA4. The fold change of GATA4 in Y-27632 treated HDFs relative to the control (expression level as 1) is shown. (**F**) HDFs were transfected with siRNAs for ROCK1 and/or ROCK2, or a scrambled siRNA as a control, and at 48 h after transfection, the mRNA expression level of GATA4 was detected by RT-qPCR analysis. The fold change of GATA4 in the ROCK knockdown HDFs relative to the control (expression level as 1) is shown. (**G**) HDFs were transfected with a scrambled siRNA (sictrl), siRNAs targeting GATA4 (siGATA4), together with either PBS or 10 μM Y-27632 or 100 ng/ml IGFBP-5 and 10 μM Y-27632 alone for 48 h, then were fixed and analyzed using a SA-β-gal staining kit to detect senescent cells, scale bar = 100 μm. (**H**) Quantification of SA-β-gal-positive cells in (**G**), the percentage of SA-β-gal-positive cells was calculated based on counting a total of 500 cells. (**I**) Proposed scheme indicating the mechanism of ROCK regulating the senescence of human skin dermal cells. All experiments were repeated at least 4 times, * P<0.05,**P<0.01, *** P<0.005, when two groups were compared as indicated, or were compared to the corresponding control.

Because IGFBP-5 has been shown to bind to and regulate the action of IGFs in multiple cellular processes including proliferation, survival, differentiation and motility, we next tested whether IGFBP-5 plays any role in regulating the cellular senescence of Y-27632 treated HDFs. First we treated HDFs with or without Y-27632 in the presence of 100 ng/ml IGFBP-5, a concentration close to what was found in the conditioned media after Y-27632 treatment. After 48 h, we analyzed the senescence of HDFs by SA-β-gal staining and found that treatment with Y-27632 and/or IGFBP-5 significantly induced the senescence of HDFs (Figure 7A, 7B). In contrast, when HDFs were treated with Y-27632 with or without the knockdown of IGFBP-5 (siIGFBP-5), which was confirmed by RT-PCR analysis (Supplementary Figure 7A), the knockdown of IGFBP-5 significantly blocked the increased cellular senescence induced by Y-27632 (Figure 7C, 7D). These results suggest that the prolonged treatment of HDFs with Y-27632 increases cellular senescence probably by enhancing the expression and production of IGFBP-5.

### IGFBP-5 expression in HDFs is regulated by the GATA4 transcription factor

We next characterized how Y-27632 regulates the expression of IGFBP-5. It has been reported that the activation of GATA4 is regulated by ROCK through the ERK/MAPK pathway [43], and that the knockdown of GATA4 decreases the expression level of IGFBP-5 in response to cAMP treatment of granulosa cells [44]. By analyzing the upstream sequence of the human *IGFBP-5* gene and scanning motifs from JASPAR (http://jaspar.genereg.net), we found a candidate motif recognized by the transcription factor GATA4 in the upstream region of the *IGFBP-5* gene. Importantly, analysis of published GATA4 CHIP-seq data (http://cistrome.org) in gastric cancer cells (AGS) [45] and in skin dermal fibroblasts [46] indicated that IGFBP-5 is a putative target of GATA4. Therefore, we investigated whether the expression of GATA4 was changed in Y-27632 treated and/or in ROCK knockdown HDFs. We found that the inhibition of ROCK, either by Y-27632 or by knockdown with ROCK1/2 siRNAs, significantly increased the expression of GATA4 (Figure 7E, 7F and Supplementary Figure 6B). Importantly, the knockdown of GATA4, which was confirmed by RT-PCR (Supplementary Figure 7B) not only blocked the senescence of HDFs, but also blocked the senescence induced by Y-27632 but did not decrease cellular senescence induced by IGFBP-5 (Figure 7G, 7H). In summary, these data suggest that Y-27632 increases the expression of GATA4, and that GATA4 then acts upstream to control the expression of *IGFBP-5* and thus the induced senescence of HDFs (Figure 7I).

## DISCUSSION

The effects of the ROCK inhibitor, Y-27632, on cells is complicated. It has impacts on the cytoskeleton [47], adhesion [10], proliferation and migration of many kinds of cells. The action of Y-27632 on cell proliferation and migration has been shown to be cell-context dependent. For instance, Y-27632 has been reported to inhibit the growth of several types of tumor cells, including melanoma cells [10, 29] and prostatic smooth muscle cells [48]. However and in contrast, our recent study showed that Y-27632 actually promotes the growth of human BRAF-mutant melanoma cells [30]. It is well-known that the addition of Y-27632 to the culture medium significantly enhances the growth of epidermal cells (keratinocytes), even in prolonged cultures [17, 31, 49]. The situation for dermal fibroblasts is less clear. While Piltti’s study showed that Y-27632 increases the proliferation of fibroblasts derived from human foreskins [33], our recent study showed that the addition of Y-27632 to initial cultures of primary skin cells actually inhibited the growth of human dermal cells [32]. To understand that discrepancy, in the present study we investigated the effects of Y-27632 on the growth of HDFs over time. Interestingly, we found that during the first 24 h, treatment with Y-27632 enhanced the growth of HDFs, as analyzed by three different approaches: counting cell numbers with an automatic cell counter (Figure 1A), performing CCK8 assays (Figure 1B) and EdU staining of proliferative cells (Figure 1C, 1D). Those results were consistent with the study of Piltti et al., in which the culture time was less than 48 h. But at later time points, i.e. 48 h and 72 h, the growth of HDFs treated with Y-27632 was significantly inhibited (Figure 1A–1D). By analyzing apoptotic and senescent cells, we discovered that the inhibition of HDF growth by Y-27632 was likely due to the increased induction of cellular senescence (Figure 2A–2C), which was further confirmed by analysis of the senescence marker Lamin B1 [42] (Supplementary Figure 3C). These results were further confirmed by the inhibition of ROCK activities using knockdown of ROCK1/2 in HDFs with siRNAs (Figure 1F, 1G). Importantly, these effects of Y-27632 were validated using cHDFs (Supplementary Figures 1, 3). These studies suggested that the inhibition of ROCK enhances HDF growth at early time points, but inhibits their growth with prolonged treatment.

To understand the potential molecular mechanism involved in the regulation of HDF growth by Y-27632, we first analyzed the expression of the classical genes involved in regulating cell proliferation and cellular senescence. We found that Y-27632 induces the expression of genes involved in proliferation (cyclinD1) and senescence (p16 and p21) and enhanced the phosphorylation of ERK and AKT at early time points (Figure 4 and Supplementary Figure 4). The activation of ERK pathways at early time points of Y-27632 treatment may contribute to the increased growth of HDFs at those early time points. Notably, the cellular senescence induced by Y-27632 was weaker initially, but became stronger with treatment time associated with decreased ERK activation, eventually leading to the cessation of HDF growth. We recognize that this result contradicts a recent study by Park et al., which reported that prolonged treatment with Y-27632 could ameliorate the senescence of one strain of foreskin-derived fibroblasts, which were used in their earlier studies published 7 years ago, in cultures in the presence of DMSO [50]. Our experiments were conducted using early passage primary HDFs without the addition of DMSO and those HDFs originated from different donors. Such different conditions could contribute to the discrepancy between our results and theirs.

To rule out processes that enhance senescence elicited by Y-27632 at the late time points, we performed RNA-seq analysis of gene expression profiles of HDFs collected after 12 h and 48 h of treatment with Y-27632. From those analyses we identified IGFBP-5, a negative regulator of cell proliferation, whose expression increased with the duration of Y-27632 treatment (a 7-fold increase after the first 24 h, Figure 6A). IGFBP-5 and other IGFBP family members have been reported to induce premature senescence in human fibroblasts and endothelial cells [51, 52] and to suppress the growth of several types of tumor cells [53–56]. We found that the addition of IGFBP-5 protein promotes the senescence of HDFs (Figure 7A, 7B), and that the senescence-inducing effect of Y-27632 was blocked by the knockdown of IGFBP-5 in HDFs (Figure 7C, 7D). Analyses of binding motifs showed that there are candidate motifs upstream in the *IGFBP-5* gene that are recognized by the transcription factor GATA4. It has been reported that the activation of GATA4 is regulated by ROCK through the ERK/MAPK pathway [43], and that the knockdown of GATA4 decreases the expression level of *IGFBP-5* in response to cAMP treatment of granulosa cells [44]. Our results show that the expression of GATA4 is increased in Y-27632-treated and ROCK1/2-knockdown HDFs (Figure 7E, 7F), suggesting that ROCK may control the expression of GATA4 in HDFs. Since we found that knockdown of GATA4 blocked the increased senescence induced by Y-27632 (Figure 7G, 7H), we infer that the Rock inhibitor induces senescence in HDFs by regulating the expression of GATA4 and IGFBP-5 (Figure 7I). Nevertheless, the detailed mechanism by which ROCK inhibition controls the level of GATA4 and IGFBP-5 still needs further clarification.

Cellular senescence is a program activated by normal cells in response to various types of stress, including telomere uncapping, DNA damage, oxidative stress, oncogene activity, mitochondrial dysfunction, reprogramming factors, wound healing, cell-cell fusion and certain cytokines [57, 58]. Changes in gene expression patterns of senescent cells are different according to the causes of senescence. Most inducers of senescence activate the tumor suppressor pathways p53/CDKN1A (p21) or CDKN2A (p16), while some rely on the activation of inflammatory transcription factors [57]. CyclinD1, a nuclear protein required for cell cycle progression in G1, has also been reported to have elevated expression levels in senescent human fibroblasts [59]. Both ERK [60] and AKT [61, 62] pathways were reported to be activated by senescent cells. Ras/Raf/ERK pathway-induced senescence correlates with the induction of senescence-associated genes, such as p16 and p21. Our results show that treatment with Y-27632 can increase the expression levels of p16, p21 and cyclinD1, and activate the ERK pathways at early time points, but the cell senescence became profound after 24 h. In light of these results, we propose that at an early time point of Y-26732 treatment, the growth signaling (indicated by cyclinD1) is likely much stronger than that of cell cycle arrest (p16 or p21) (Figure 4). However, after 12 h of treatment with Y-26732, the dramatic induction of IGFBP-5 by Y-27632 (Figure 6) resulted in the promotion of senescence. Therefore, we think that the increased senescence of HDFs elicited by Y-27632 is mainly due to the increased expression of IGFBP-5, which may be independent of p16/p21.

Previous studies have shown that that the senescence of stromal cells can be associated with the induction of CAF effector genes [63], and the senescent fibroblasts are able to release cytokines and growth factors into their microenvironment, termed “a senescence-associated secretory phenotype (SASP)”. That process converts senescent fibroblasts into a CAF phenotype, i.e. cells with the ability to promote tumor development and progression [41]. The present study demonstrated that prolonged treatment with Y-27632 can enhance the expression of CAF effector genes in HDFs and that the senescent HDFs obtain a CAF-like phenotype (Figure 3). Recent studies demonstrated that stromal cells can take on a pro-carcinogenic state independent of the presence of tumor cells, and can alter the stromal microenvironment [63, 64]. It has been shown that the Ki values of Y-27632 for ROCK1 and ROCK2 are 0.22 μM and 0.3 μM, respectively, and that Y-27632 at concentrations of 0.03-0.6 μM significantly inhibit Rho-kinase activity *in vivo* [5, 20, 65], which are much lower than the concentrations used in this study and in most other studies. Therefore, we also treated HDFs with these low concentrations of Y-27632, and found that 2 μM Y-27632 increases the cellular senescence of HDFs, while 0.15 and 0.3 μM Y-27632 didn’t. However, both the 0.15 and 0.3 μM concentrations of Y-27632 induced changes in the expression of p16, IGFBP-5 and GATA4 (Supplementary Figure 8), indicating the potential effects on HDFs. In a clinical trial, the short-term efficacy and safety of ROCK inhibitors in the treatment of pulmonary hypertension has been demonstrated to be effective for decreasing pulmonary artery pressure without apparent side effects, however the long-term efficacy and safety of ROCK inhibitors needs to be clarified [18]. Moreover, ROCK inhibitors have been proposed to be developed as therapeutic compounds for treating various diseases including cancers [66–69]. Therefore, it will be important to establish if such prolonged treatment with Y-27632 can induce the senescence of HDFs and eventually has the potential to transform primary dermal fibroblasts into CAFs.

In summary, the present study demonstrates for the first time that Y-27632 can promote cellular senescence in human skin primary fibroblasts by inducing the expression of IGFBP-5. Our results indicate that prolonged treatment with Y-27632 may eventually lead to the transformation of human skin fibroblasts to CAFs. These findings have important implications for the clinical application of ROCK inhibitors.

## MATERIALS AND METHODS

### Isolation and culture of primary HDFs

The derivation of primary HDFs from adult foreskins, and their isolation and culture were performed according to our previous publication [32]. Briefly, each foreskin was cut and incubated in dispase (2.5 mg/ml) solution overnight at 4°C. The next day, the dermis was separated from the epidermis, then minced and incubated with type I collagenase (2.5 mg/mL) at 37°C for 1 h. The digestion was stopped with Dulbecco’s modified Eagle’s medium (DMEM, Gibco, Waltham, MA, United States) containing 10% fetal bovine serum (FBS), and the cell solutions were filtered and centrifuged to obtain the cell pellets. The cell pellets were washed once with phosphate buffered saline (PBS) and then resuspended in DMEM with 10% FBS for initial culture (P0). After the initial culture, the HDFs were cloned by limiting dilution. The cloned HDFs were validated by immunofluorescence staining to be positive for the mesenchymal cell marker vimentin and negative for the epidermal cell marker pan-CK. The cloned HDFs were then expanded in culture and no significant changes in morphology or growth were observed after at least 10 passages. Three independent HDF clones were derived from three different donors. In order to minimize the effects of passaging cells, such as inducing differentiation or senescence, we used low passages of HDFs, which allowed 3 or 4 passages to obtain a sufficient number of cells, for all experiments. Y-27632 (Sigma-Aldrich, St. Louis, MO, United States) was dissolved in PBS (phosphate-buffered saline) to prepare a 10 mM stock solution and was kept in a -20°C freezer. For treatment, the stock solution was added into the culture medium at final concentrations of 0.15, 0.3, 2 or 10 μM. Ten μM Y-27632, the concentration used in most *in vitro* studies [10, 70] was used for most experiments in the present study, and for the control group, the same volume of PBS was added. Commercial adult human dermal fibroblasts (cHDFs) were purchased from ScienCell (Cat. 2320, Carlsbad, CA, United States) and were used to validate the effects of Y-27632 observed for our own cloned HDFs. The same growth conditions were used to expand cHDFs in culture, and cHDFs at passages 3 or 4 were used for all experiments.

### Cell number counting

HDFs were plated in 35 mm dishes and incubated in DMEM containing 10% FBS with or without 10 μM Y-27632. The cells were detached by trypsinization at 12, 24, 48 and 72 h after plating and were exposed to 0.1% trypan blue. The number of trypan blue negative cells was counted using a microscopic counting chamber.

### Cell proliferation assays

Cell proliferation assays were carried with the following two approaches:

Cell Counting Kit-8 (CCK 8, Dojindo, Kumamoto, Japan) assay: A total of 5 × 10^3^ HDFs were seeded into 96-well plates with or without 10 μM Y-27632. Cell viability was measured using the CCK-8 solution reagent at the times indicated in the Figures. Ten μl CCK-8 solution were added per well, and the plates were incubated at 37°C for 2 h, after which the absorbance was measured at 450 nm.

EdU Cell Proliferation assay: 5-ethynyl-2'-deoxyuridine (EdU) staining was conducted using the BeyoClick™ EdU Cell Proliferation Kit with Alexa Fluor 594 (Beyotime, Shanghai, China). The cells were incubated with 10 μM EdU for 2 h at 37°C and 5% CO_2_, and then fixed in 4% paraformaldehyde at room temperature for 30 min before being stained with 4’,6-Diamidine-2’-phenylindole dihydrochloride (DAPI) for 3 min. After an additional wash in PBS, the cells were observed using an inverted fluorescence microscope.

### Cell apoptosis assay

Cells were seeded at 5×10^4^ cells per well in six-well plates with or without Y-27632. After culture for 48 h, apoptosis measurements were performed using flow cytometry analysis with an Annexin V-FITC Apoptosis Detection Kit (Beyotime, Shanghai, China). Cells were stained with 5 μl Annexin V-FITC and 10 μl propidium iodide (PI) for 15 min and were then analyzed by flow cytometry.

### RNA extraction and reverse transcription- quantitative polymerase chain reaction (RT-qPCR)

Total RNAs were extracted from HDFs using Trizol reagent (TaKaRa, Shiga, Japan), according to the manufacturer's instructions. Purified RNAs were reverse-transcribed to cDNAs using Prime Script RT-polymerase (TaKaRa, Shiga, Japan), followed by RT-qPCR in a LightCycler 480 Real-Time PCR system (Roche Diagnostics, Rotkreuz, Switzerland). The mRNA levels of all detected genes were first normalized to that of the internal control human housekeeping gene 36B4, then the expression level (fold-change) of detected genes at different conditions were calculated relative to that of the corresponding control group (expression level as 1). Oligo sequences for all primers are listed in Supplementary Table 1.

### RNA-sequencing and analysis

Total RNAs were isolated from dermal cells with or without Y-27632 treatment for 12 h or 48 h, after which mRNAs were purified from each total RNA using poly-T oligo-attached magnetic beads. After sequencing libraries were prepared using NEBNext® UltraTM RNA Library Prep Kit for Illumina® (New England Biolabs, Beverly, MA, United States), the library preparations were sequenced using an Illumina Hi seq platform and 125 bp/150 bp paired-end reads were generated. Genes with adjusted P values <0.05 found by DESeq2 were assigned as differentially expressed genes (DEGs). Gene Ontology (GO) enrichment analysis of DEGs was implemented by the cluster Profiler R package, in which gene length bias was corrected. GO terms with corrected P values <0.05 were considered significantly enriched for DEGs.

### β-Galactosidase staining for detection of cellular senescence

HDFs were plated in 24-well plates for the indicated times and were then fixed with 4% formaldehyde. Senescence-associated β-galactosidase (SA-β-gal) was assayed using a senescence β-Galactosidase Staining Kit (Beyotime, Shanghai, China). Images of five randomly selected fields from each well were analyzed with an Olympus IX71 digital microscope. At least 300 cells for each condition were analyzed, and the data were obtained from a minimum of three independent experiments.

### Small interfering RNA (siRNA) assays

Transfection of siRNAs into HDFs was performed as previously described [71]. Briefly, HDFs were seeded in 6-well plates, grown to 70-80% confluence, and then transfected with 20 nM siRNA using lipofectamine 3000 (Invitrogen, Carlsbad, United States). Forty-eight h later, cells were collected for RT-PCR and western-blot analyses to determine the knockdown efficiency. siRNA oligos that target *ROCK1*, *ROCK2*, *IGFBP-5* and *GATA4* genes or a negative control scramble siRNA were synthesized by Shanghai Shenggong Company (Shanghai, China). Three independent siRNAs for each gene were tested for knockdown efficiency. Oligo sequences of all siRNAs used in this work are listed in Supplementary Table 2.

### Enzyme-linked Immuno-Sorbent Assays (ELISA)

HDFs were incubated under different conditions as indicated in the Figures and cell culture supernatants were collected at the indicated times. The amounts of IGFBP-5, TNC or PDGFRα in the supernatant fluids were determined using ELISA Kits (Abcam, Cambridge, United Kingdom) according to the manufacturer’s instructions.

### Western – blotting analysis

Cell protein lysates were separated by 10% SDS-PAGE and were electrophoretically transferred to polyvinylidene difluoride (PVDF) membranes (Millipore, St. Louis, MO, United States). After incubation with specific monoclonal antibodies or with a GAPDH specific antibody used as a loading control, the membranes were incubated with horseradish peroxidase (HRP)-labeled goat-anti-mouse or goat-anti-rabbit IgG (Cell Signaling Technology, Beverly, MA, United States) at room temperature for 1 h according to the manufacturer’s instructions. Signal detection was carried out using an ECL system (Amersham Pharmacia, Piscataway, NJ, United States). Immunoblotting was performed using the following primary antibodies: ROCK1 (sc-17794, Santa Cruz Biotechnology, Santa Cruz, CA, United States), ROCK2 (sc-5561, Santa Cruz), p16 (ab-51243, Abcam), p21 (2946, Cell Signaling Technology), CYCLIND1 (2978, Cell Signaling Technology), pERK1/2 (Thr202/Tyr204) (4370, Cell Signaling Technology), total ERK1/2 (4695, Cell Signaling Technology), pAKT (ser473) (4060, Cell Signaling Technology), total AKT (4691, Cell Signaling Technology), tubulin (Cell Signaling Technology) and GAPDH (Cell Signaling Technology). To quantify western-blot assays, the band densities normalized by the housekeeping gene GAPDH were quantified using ImageJ software (NIH, Rockville, MD, United States), and the fold change of detected proteins in Y-27632 treated HDFs relative to the corresponding control cells was calculated (expression level as 1).

### Y-27632 *in vivo* treatment

The culture procedure of primary human epidermal cells, isolated from adult foreskin tissues, exactly followed our previous publications [32, 72], and human reconstituted skin was generated also according to what we previously described [72, 73]. Briefly, 3 x 10^6^ passage 3 HDFs mixed with 2 x 10^6^ passage 3 human epidermal cells were grafted onto 1 cm x 1 cm open wounds, which were created in the dorsal skin of 8-week old female nude/nude mice (Charles River Laboratory, Wilmington, MA, USA). At 1 month after grafting, 10 μl of 10 mM Y-27632 was injected subcutaneously into the reconstructed skin once every two days for 2 weeks. The control group was injected with PBS, with 4 mice for each group. At 2 weeks after treatment, the reconstituted skins were collected and the dermis was immediately separated from the epidermis using a dissection microscope. The separated dermis was directly processed for RNA extraction to perform RT-qPCR analysis of IGFBP-5 expression.

### Statistical analysis

All experiments were repeated at least 3 times (n=3) with HDFs derived from three different donors. Data are expressed as means ± standard deviation (mean ± SD), p values from the statistical analyses are indicated in the Figures. Student’s t-test was used to analyze differences between two experimental groups. One-way or two-way ANOVA with correction for multiple pairwise comparisons was used when comparing more than 2 groups with one or two independent variables, respectively.

### Ethics statement

Adult foreskin tissues without any personal identity information were obtained from participants who had provided verbal and written informed consent at Qilu Hospital, Shandong University. The procedure for obtaining foreskin tissues was approved by the Medical Ethical Committee of the School of Stomatology, Shandong University (Protocol No. GR201711, Date: 02-27-2017).

The animal protocol was approved by the Ethics Committee of the Hospital of Stomatology, Shandong University (Protocol No. GR201720, Date: 02-27-2017). All animal procedures in this study followed National Institutes of Health Guidelines for the Care and Use of Laboratory Animals and the principles of the Basel Declaration.

### Data availability statement

The data that support the findings of this study are available from the corresponding author upon reasonable request. The RNA-seq data have been submitted to the GEO database under the accession code: GSE135934.

## Supplementary Material

Supplementary Figures

Supplementary Tables
