# Supplementary material for: Prolonged treatment with Y-27632 promotes the senescence of primary human dermal fibroblasts by increasing the expression of IGFBP-5 and transforming them into a CAF-like phenotype
Source: Aging (Albany NY). 2020 Aug 25;12(16):16621–46. doi: 10.18632/aging.103910 (PMC7485707; doi:10.18632/aging.103910)
Supplement: Supplementary Figures [file aging-12-103910-s002..pdf]

SUPPLEMENTARY FIGURES

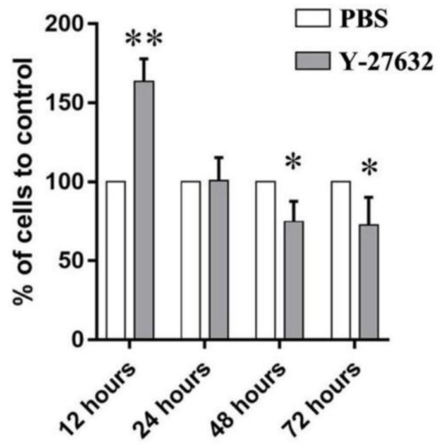

Supplementary Figure 1. Prolonged treatment with Y-27632 inhibits the growth of commercial HDFs. Commercial HDFs were cultured under the same conditions as described for Figure 1A, and cells were collected at the indicated times for analysis of cell proliferation using a CCK8 kit; the percentage of cell growth was calculated relative to the corresponding control (PBS) as 100.

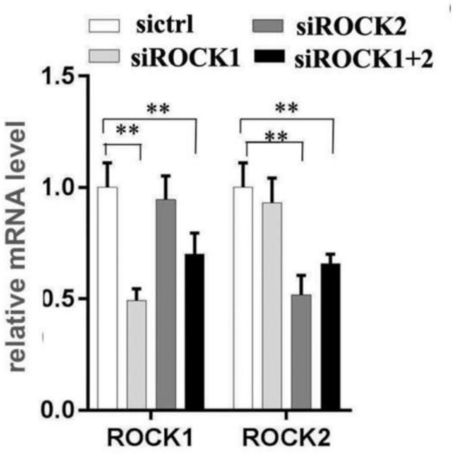

Supplementary Figure 2. ROCK1/2 expression was efficiently knocked down in HDFs by transfection of the corresponding siRNAs. HDFs were transfected with siRNAs specific for ROCK1 (siROCK1), ROCK2 (siROCK2) or both ROCK1 and ROCK2 (siROCK1+2); control HDFs were transfected with a scrambled siRNA (sictrl), and cells were collected at 48 h after transfection to detect the expression of ROCK1 and ROCK2 by RT-qPCR analysis.

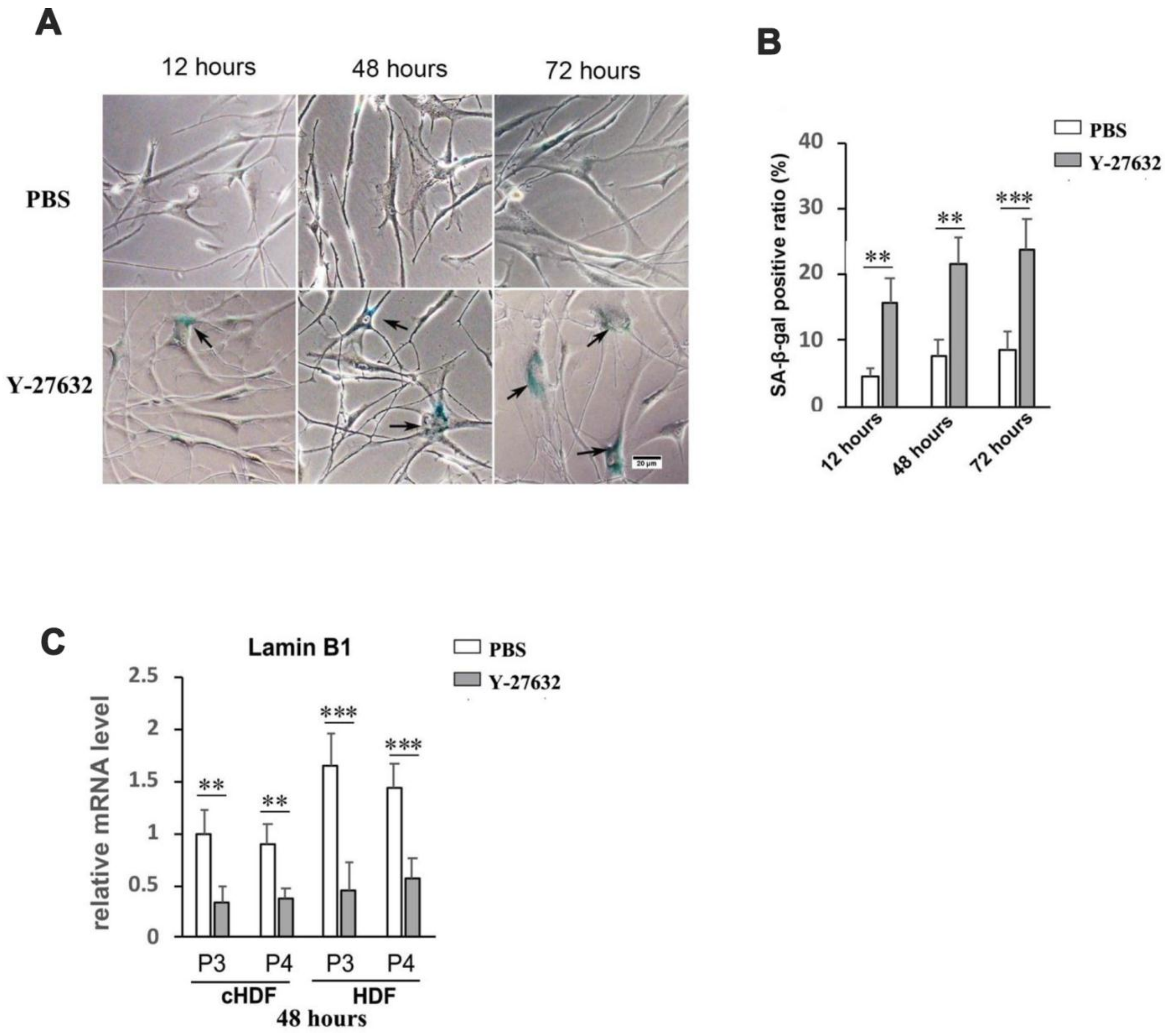

Supplementary Figure 3. Prolonged treatment with Y-27632 enhances the senescence of commercial HDFs. (**A**, **B**) Commercial HDFs (cHDFs) were treated with PBS or 10 μM Y-27632, and then were fixed at the indicated times and analyzed using a SA-β-gal staining kit to detect senescent cells. scale bar = 20 μm. (**B**) Quantification of (**A**), the percentage of SA-β-gal-positive cells (arrows, blue) was calculated based on counting a total of 500 cells. (**C**) Passage 3 or passage 4 commercial HDFs (cHDFs) and our own cloned HDFs (HDFs) were treated with PBS or 10 μM Y-27632 for 48 h, after which they were collected for analysis of lamin B1 expression by RT-qPCR analysis.

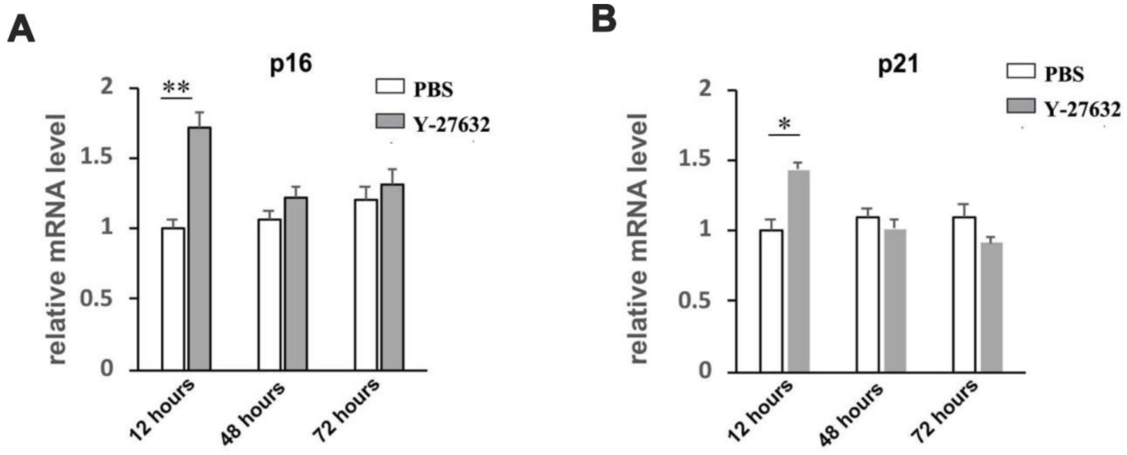

Supplementary Figure 4. Y-27632 enhances expression of senescence markers p16 and p21 in commercial HDFs at 12 h after treatment. (**A**, **B**). Commercial HDFs were treated with PBS or 10 μM Y-27632, then were collected at the indicated times for analysis of p16 and p21 mRNA expression levels by RT-qPCR. All experiments were repeated at least 3 times, *P<0.05 , **P<0.01, ***P<0.005 when compared to the corresponding control as indicated.

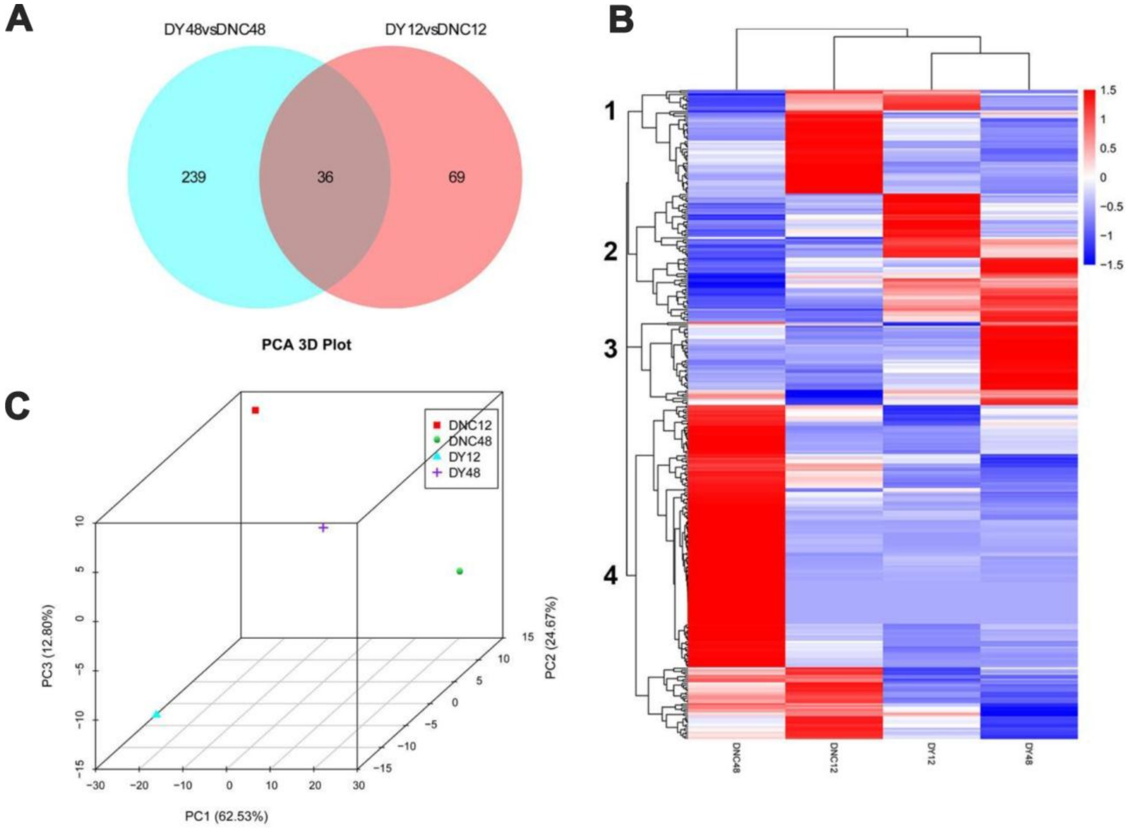

Supplementary Figure 5. RNA-seq analysis of different gene expression profiles and related pathways of HDFs treated with or without Y-27632. (**A**) The number of DEGs in HDFs treated with Y-27632 at 12 h (DY12) and 48 h (DY28) versus their corresponding controls (DNC12 and DNC48); 36 of those DEGs appeared at both time points. (**B**) All DEGs are shown in a heatmap and were divided into four clusters as indicated. DEGs in cluster 1 were downregulated both in Y+ and Y- at 12 h. DEGs in cluster 2 were downregulated only in both Y-27632 treated groups. DEGs in cluster 3 were upregulated only after Y-27632 treatment at 48 h. DEGs in cluster 4 were downregulated after Y-27632 treatment at 12 h and 48 h. (**C**) Principal component analysis (PCA) of the 4 groups based on the expression of all DEGs.

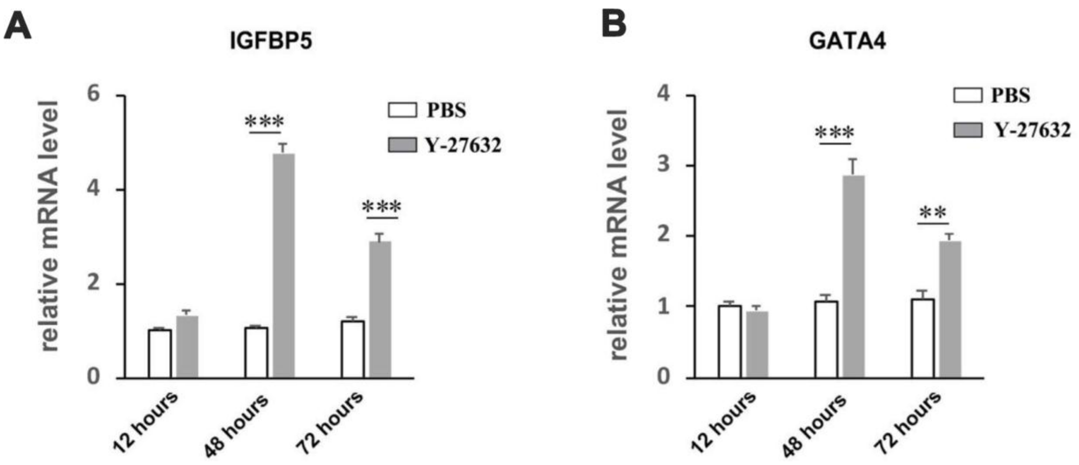

Supplementary Figure 6. Prolonged treatment with Y-27632 induces the expression of IGFBP-5 and GATA4 in commerical HDFs. (**A**, **B**) Commercial HDFs were treated with PBS or 10 μM Y-27632, then were collected at the indicated times for analysis of IGFBP-5 and GATA4 mRNA expression levels by RT-qPCR. All experiments were repeated at least 3 times, **P<0.01, ***P<0.005 when compared to the corresponding control as indicated.

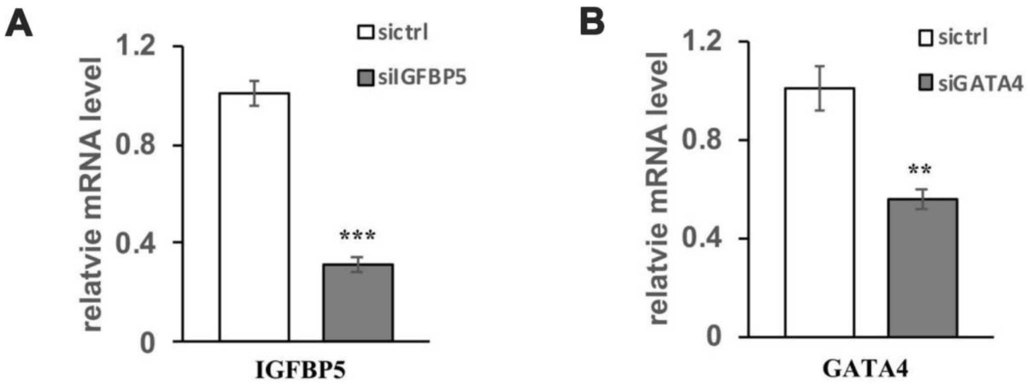

Supplementary Figure 7. IGFBP-5 and GATA4 was knocked down efficiently by their corresponding siRNAs. (**A**, **B**) HDFs were transfected with siRNAs, which were mixed with 3 independent siRNAs, targeting either IGFBP-5 or GATA4 and with a scrambled siRNA as a control (sictrl) and 48 h after transfection, mRNA levels of IGFBP-5 or GATA4 were analyzed using RT-qPCR. The fold change of mRNA levels in the siRNA-transfected HDFs relative to control HDFs (expression level as 1) is shown. All experiments were repeated at least 3 times, **P<0.01, ***P<0.005 when compared to the corresponding control as indicated.

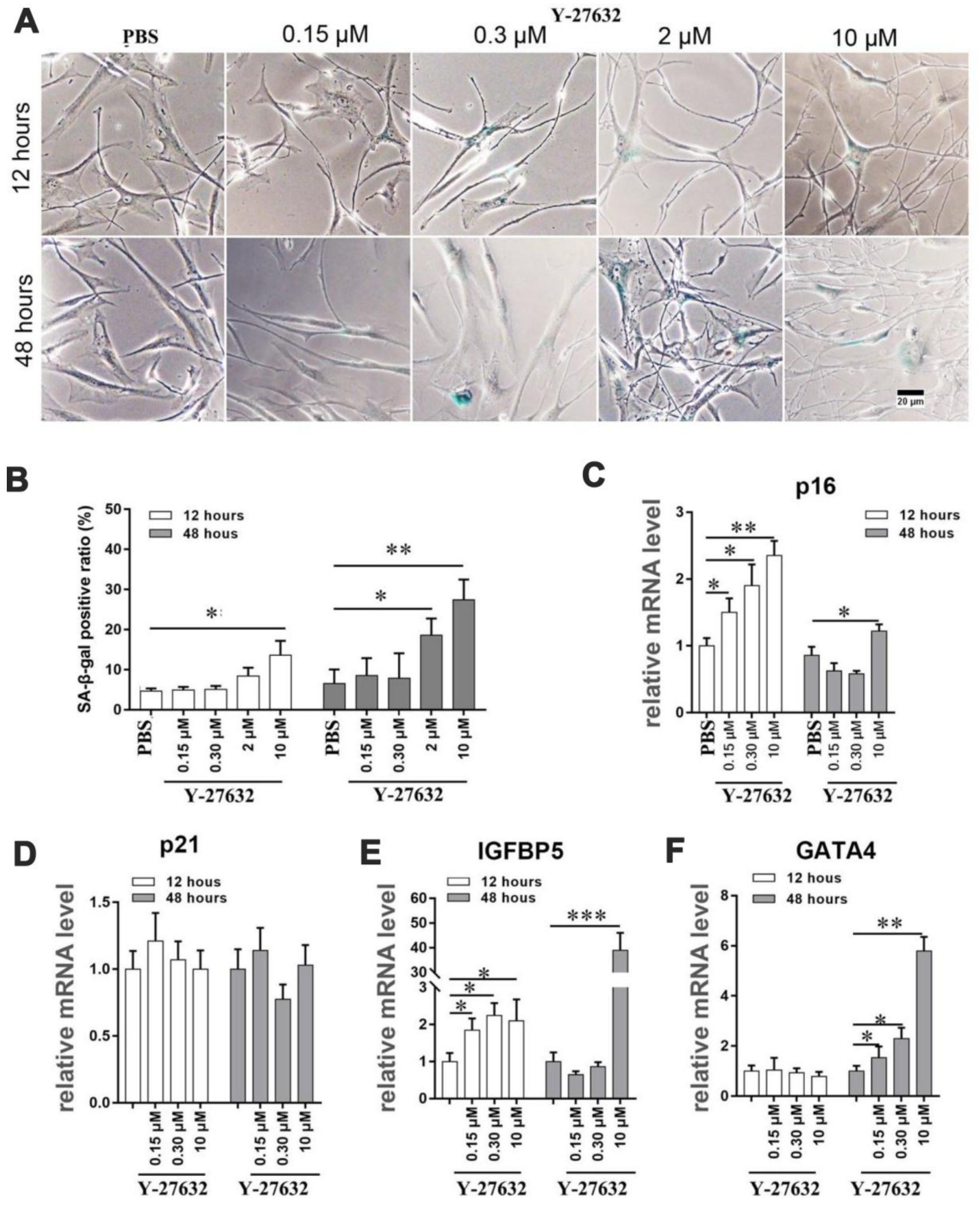

Supplementary Figure 8. Low concentrations of Y-27632 don't enhance cellular senescence in HDFs, but do induce the expression of p21, IGFBP-5 and GATA4. (**A**, **B**) HDFs were treated with different concentrations of Y-27632 as indicated or PBS2 as a control, and were fixed at the indicated times and analyzed using a SA-β-gal staining kit to detect senescent cells. scale bar = 20 μm. (**b**) Quantification of (**a**), the percentage of SA-β-gal-positive cells (blue) was calculated based on counting a total of 500 cells. (**C**–**F**) HDFs were treated with different concentrations of Y-27632 as indicated or PBS as a control, and cells were collected at 12 and 48 h to analyze mRNA expression levels of p16, p21, IGFBP-5 and GATA4 by RT-qPCR. All experiments were repeated at least 3 times, *P<0.05 , **P<0.01, ***P<0.005 when compared to the corresponding control as indicated.
