# Supplementary material for: Prolonged treatment with Y-27632 promotes the senescence of primary human dermal fibroblasts by increasing the expression of IGFBP-5 and transforming them into a CAF-like phenotype
Source: Aging (Albany NY). 2020 Aug 25;12(16):16621–46. doi: 10.18632/aging.103910 (PMC7485707; doi:10.18632/aging.103910)
Supplement: Supplementary Tables [file aging-12-103910-s001..pdf]

SUPPLEMENTARY TABLES

Supplementary Table 1. Primer sequences used for quantitative real-time PCR (qRT-PCR).

Primer	Sequences (5'-3')	Primer	Sequences (5'-3')
**ROCK1 forward**	TGCCTTCCTTACTGACAGGG	**ACTA2 forward**	ATCCCCGGGACTAAGACGG
**ROCK1 reverse**	CCAAGCCCACTGGTCATTTT	**ACTA2 reverse**	AGAGCCATTGTCACACACCA
**ROCK2 forward**	GCACAGTTTGAGAAGCAGCT	**MMP1 forward**	CCAGGTATTGGAGGGGATGC
**ROCK2 reverse**	TACCACGCTTGACAGGTTCT	**MMP1 reverse**	GTCCAAGAGAATGGCCGAGT
**CYCLIND1 forward**	CCCTCGGTGTCCTACTTCAAA	**TNC forward**	AGCATCCGGACCAAAACCAT
**CYCLIND1 reverse**	AAGACCTCCTCCTCGCACTT	**TNC reverse**	CCGATGCCATCCAGGAAACT
**P16 forward**	TAGTTACGGTCGGAGGCCG	**PDGFRl forward**	CTGAGGTTCAGAGCGCGG
**P16 reverse**	ACGGGTCGGGTGAGAGTG	**PDGFR1 reverse**	CACCTCCACCAAGTCCTCG
**P21 forward**	AGTCAGTTCCTTGTGGAGCC	**Serpine 1 forward**	GAAAGGCAACATGACCAGGC
**P21 reverse**	GCATGGGTTCTGACGGACAT	**Serpine 1 reverse**	ACATGTCGGTCATTCCCAGG
**GATA4 forward**	GTGTCCCAGACGTTCTCAGTC	**Desmin forward**	AATGACCGCTTCGCCAACTA
**GATA4 reverse**	GGGAGACGCATAGCCTTGT	**Desmin reverse**	GGTTAGTGAGCACCTCCACC
**IGFBP-5 forward**	ACGAAAAGAGCTACCGCGAG	**Vimentin forward**	AATGGCTCGTCACCTTCGTG
**IGFBP-5 reverse**	GAGTAGGTCTCCTCGGCCAT	**Vimentin reverse**	CAGAGAAATCCTGCTCTCCTCG
**IGFBP 1 forward**	ATCCTTTGGGACGCCATCAG	**FSP-1 forward**	GTACTCGGGCAAAGAGGGTG
**IGFBP 1 reverse**	AGAGTTCTATTCGGCAGGGC	**FSP-1 reverse**	TTGTCCCTGTTGCTGTCCAA
**HNF forward**	GGACATGGCCGACTACAGTG	**Lamin B1 forward**	AAGCAGCTGGAGTGGTTGTT
**HNF reverse**	CGTTGAGGTTGGTGCCTTCT	**Lamin B1 reverse**	TTGGATGCTCTTGGGGTTC
**NKD1 forward**	ACTTTCGGCTGGAAGTGGC	**H36B4 forward**	GCAATGTTGCCAGTGTCTGT
**NKD1 reverse**	CAGGGTTCGCTCACTCTCTC	**H36B4 reverse**	GCCTTGACCTTTTCAGCAAG

Supplementary Table 2. Oligo sequences used for siRNA.

siRNA	Sequences (5'-3')
ROCK1 sense	GCAGAUGAAACAGGAAAUATT
ROCK1 antisense	UAUUUCCUGUUUCAUCUGCTT
ROCK2 sense	GCAGCUGGAAUCUAACAAUTT
ROCK2 antisense	AUUGUUAGAUUCCAGCUGCTT
IGFBP-5 01 sense	GCUGACCCAGUCCAAGUUUTT
IGFBP-5 01 antisense	AAACUUGGACUGGGUCAGCTT
IGFBP-5 02 sense	GCUGUGUACCUGCCCAAUUTT
IGFBP-5 02 antisense	AAUUGGGCAGGUACACAGCTT
IGFBP-5 03 sense	GGACAAGUACGGGAUGAAGTT
IGFBP-5 03 antisense	CUUCAUCCCGUACUUGUCCTT
GATA4 01 sense	GGCAGAGAGUGUGUCAACUTT
GATA4 01 antisense	AGUUGACACACUCUCUGCCTT
GATA4 02 sense	GCCCAAGAACCUGAAUAAATT
GATA4 02 antisense	UUUAUUCAGGUUCUUGGGCTT
GATA4 03 sense	UCAACCGGCCGCUCAUCAATT
GATA4 03 antisense	UUGAUGAGCGGCCGGUUGA
Negative control sense	UUCUCCGAACGUGUCACGUTT
Negative control antisense	ACGUGACACGUUCGGAGAATT
